# Hand dexterity, daily functioning and health-related quality of life in kidney transplant recipients

**DOI:** 10.1038/s41598-022-19952-5

**Published:** 2022-09-28

**Authors:** Tim J. Knobbe, Daan Kremer, Michele F. Eisenga, Eva Corpeleijn, Coby Annema, Joke M. Spikman, Transplantlines Investigators, Coby Annema, Stephan J. L. Bakker, Stefan P. Berger, Hans Blokzijl, Frank Bodewes, Marieke T. de Boer, Kevin Damman, Martin H. de Borst, Arjan Diepstra, Gerard Dijkstra, Rianne M. Douwes, Michele F. Eisenga, Michiel E. Erasmus, C. Tji Gan, Antonio W. Gomes Neto, Heleen Grootjans, Eelko Hak, M. Rebecca Heiner-Fokkema, Bouke G. Hepkema, Frank Klont, Tim J. Knobbe, Daan Kremer, Henri G. D. Leuvenink, Willem S. Lexmond, Vincent E. de Meijer, Hubert G. M. Niesters, L. Joost van Pelt, Robert J. Pol, Robert J. Porte, Adelita V. Ranchor, Jan Stephan F. Sanders, Joëlle C. Schutten, Marion J. Siebelink, Riemer J. H. J. A. Slart, J. Casper Swarte, Wim Timens, Daan J. Touw, Marius C. van den Heuvel, Coretta C. van Leer-Buter, Marco van Londen, Erik A. M. Verschuuren, Michel J. Vos, Rinse K. Weersma, Gerjan Navis, Stefan P. Berger, Stephan J. L. Bakker

**Affiliations:** 1 Department of Internal Medicine, Division of Nephrology, University Medical Center Groningen, University of Groningen, 9700 RB Groningen, The Netherlands; 2 Department of Epidemiology, University Medical Center Groningen, University of Groningen, 9700 RB Groningen, The Netherlands; 3 Department of Health Sciences, Section of Nursing Science, University Medical Center Groningen, University of Groningen, 9700 RB Groningen, The Netherlands; 4 Department of Neurology, Division of Neuropsychology, University Medical Center Groningen, University of Groningen, 9700 RB Groningen, The Netherlands; 5 Department of Internal Medicine, Division of Nephrology, University Medical Center Groningen, Hanzeplein 1, P.O. Box 30.001, 9713 GZ Groningen, The Netherlands; 6 Department of Gastroenterology and Hepatology, University Medical Center Groningen, University of Groningen, 9700 RB Groningen, The Netherlands; 7 Department of Pediatrics, Division Pediatric Gastroenterology and Hepatology, University Medical Center Groningen, University of Groningen, 9700 RB Groningen, The Netherlands; 8 Department of Surgery, Division of Hepato-Pancreato-Biliary Surgery and Liver Transplantation, University Medical Center Groningen, University of Groningen, 9700 RB Groningen, the Netherlands; 9 Department of Cardiology, University Medical Center Groningen, University of Groningen, 9700 RB Groningen, the Netherlands; 10 Department of Pathology & Medical Biology, University Medical Center Groningen, University of Groningen, 9700 RB Groningen, The Netherlands; 11 Department of Cardiac Surgery, University Medical Center Groningen, University of Groningen, 9700 RB Groningen, the Netherlands; 12 Department of Respiratory Diseases, Tuberculosis and Lung Transplantation, University Medical Center Groningen, 9700 RB Groningen, The Netherlands; 13 Unit of PharmacoTherapy, -Epidemiology and -Economics, Groningen Research Institute of Pharmacy, University Medical Center Groningen, University of Groningen, 9700 RB Groningen, The Netherlands; 14 Laboratory of Metabolic Diseases, University Medical Center Groningen, University of Groningen, 9700 RB Groningen, the Netherlands; 15 Department of Laboratory Medicine, Transplantation Immunology, University Medical Center Groningen, University of Groningen, 9700 RB Groningen, The Netherlands; 16 Department of Clinical Pharmacy and Pharmacology, University Medical Center Groningen, University of Groningen, 9700 RB Groningen, The Netherlands; 17 Department of Surgery, Surgical Research Laboratory, University Medical Center Groningen, University of Groningen, 9700 RB Groningen, the Netherlands; 18 Department of Medical Microbiology and Infection Prevention, Division of Clinical Virology, University Medical Center Groningen, University of Groningen, 9700 RB Groningen, The Netherlands; 19 Department of Laboratory Medicine, University Medical Center Groningen, University of Groningen, 9700 RB Groningen, The Netherlands; 20 Department of Surgery, Division of Vascular Surgery, University Medical Center Groningen, University of Groningen, 9700 RB Groningen, The Netherlands; 21 Department of Health Psychology, University Medical Center Groningen, University of Groningen, 9700 RB Groningen, The Netherlands; 22 University Medical Center Groningen Transplant Center, University Medical Center Groningen, University of Groningen, 9700 RB Groningen, The Netherlands; 23 Medical Imaging Centre, Department of Nuclear Medicine and Molecular Imaging (EB50), University Medical Center Groningen, University of Groningen, 9700 RB Groningen, The Netherlands; 24 Department of Pharmaceutical Analysis, Groningen Research Institute of Pharmacy, University of Groningen, 9700 RB Groningen, The Netherlands; 25 Department of Virology, University Medical Center Groningen, University of Groningen, 9700 RB Groningen, The Netherlands

**Keywords:** Quality of life, Nephrology

## Abstract

Impaired interplay between sensory and motor function may be an important, often overlooked cause of the decreased daily functioning and impaired health-related quality of life (HRQoL) of kidney transplant recipients (KTR). We assessed this interplay using a hand dexterity test, and investigated its potential associations with daily functioning and HRQoL among KTR enrolled at the TransplantLines Biobank and Cohort Study. A total of 309 KTR (58% male, mean age 56 ± 13 years) at median 4 [IQR: 1–11] years after transplantation were included. Impaired hand dexterity, as defined by a test performance slower than the 95th percentile of an age- and sex-specific reference population, was observed in 71 (23%) KTR. Worse hand dexterity was independently associated with worse performance on almost all measures of physical capacity, activities of daily living and societal participation. Finally, hand dexterity was independently associated with physical HRQoL (standardized beta − 0.22, 95%CI − 0.34 to − 0.09, *P* < 0.001). In conclusion, impaired interplay between sensory and motor function, as assessed by hand dexterity, is prevalent among KTR. In addition, poor hand dexterity was associated with impaired daily functioning and limited physical HRQoL. Impaired interplay between sensory and motor function may be therefore an important, hitherto overlooked, phenomenon in KTR.

## Introduction

Health-related quality of life (HRQoL) of kidney transplant recipients (KTR) remains low compared to the general population, despite successful transplantation^1^. This is in part attributable to limitations in daily functioning, manifesting as impairments in physical capacity, lower ability to perform activities of daily living (ADL) and reduced societal participation^1–4^. A prerequisite for optimal functioning in these different areas is adequate functioning of the central and peripheral nervous system. Although history of kidney failure with or without dialysis and immunosuppressive therapy are known to damage the neurological system, the interplay between the sensory and motor function of the nervous system has remained largely unexplored among KTR^5–7^.

Hand dexterity, the ability to make accurate and coordinated finger and hand movements in order to manipulate objects, leans heavily on adequate interplay between sensory and motor function. Hence, hand dexterity may be a sensitive indicator of nervous system damage caused by kidney disease and treatment. Hand dexterity can be objectively measured with tests. These tests do not only tap into sensory-motor skills, but also require motor planning, visual search and focused attention^8–10^. Hand dexterity tests may therefore be a reflection of someone’s ability to perform coordinated movements and attentional control, both essential for physical activities, ADL and societal participation, since these skills are essential for these tasks^11^.

Hand function itself is also important, since it is required for performance of many tasks in daily life, and is likely crucial for societal participation: the hands are needed for almost all jobs, leisure activities, and for making and maintaining social connections with people using smartphones and computers. However, to best of our knowledge, the interplay between sensory and motor function, as assessed by hand dexterity, has not yet been investigated in KTR, and its associations with daily functioning and HRQoL among KTR are unknown. In this study, we therefore aimed to assess hand dexterity among KTR, and to identify potential clinical or biochemical determinants of hand dexterity. In addition, we investigated associations of hand dexterity with daily functioning and HRQoL.

**Table 1:** Baseline characteristics and associations with hand dexterity.

	Total population *N* = 309	Results of linear regression analyses			
		Univariable analyses		Analyses adjusted for sex and age	
		St. β (95% CI)	*P*	St. β (95% CI)	*P*
Demographics					
Male sex, *n* (%)	180 (58)	0.29 (0.18 to 0.40)	< 0.001	–	–
Age, years	56 ± 13	0.44 (0.34 to 0.54)	< 0.001	–	–
Educational level, *n* (%)					
Low	114 (38)	Reference	n/a	Reference	n/a
Medium	114 (38)	− 0.21 (− 0.33 to − 0.08)	0.001	− 0.09 (− 0.20 to 0.02)	0.1
High	304 (25)	− 0.20 (− 0.33 to − 0.08)	0.001	− 0.18 (− 0.29 to − 0.07)	< 0.001
Caucasian, *n* (%)	297 (4)	− 0.08 (− 0.19 to 0.04)	0.2	− 0.08 (− 0.17 to 0.02)	0.1
Body mass index, kg/m^2^	28 ± 5	0.07 (− 0.04 to 0.18)	0.2	0.11 (0.01 to 0.21)	0.025
Primary kidney disease, *n* (%)					
Unknown	46 (15)	Reference	n/a	Reference	n/a
Inflammatory disease	103 (33)	0.02 (− 0.14 to 0.18)	0.8	0.03 (− 0.11 to 0.16)	0.7
Congenital and hereditary kidney disease	83 (27)	− 0.13 (− 0.28 to 0.03)	0.1	− 0.13 (− 0.26 to 0.01)	0.062
Kidney vascular disease, excl. vasculitis	27 (9)	0.02 (− 0.11 to 0.15)	0.8	− 0.02 (− 0.13 to 0.10)	0.8
Diabetic kidney disease	20 (7)	0.17 (0.04 to 0.30)	0.010	0.17 (0.06 to 0.28)	0.003
Other	30 (10)	− 0.02 (− 0.15 to 0.12)	0.8	− 0.03 (− 0.14 to 0.09)	0.6
Diabetes, *n* (%)	86 (28)	0.22 (0.11 to 0.33)	< 0.001	0.17 (0.08 to 0.27)	< 0.001
Anemia, *n* (%)	96 (31)	0.08 (− 0.03 to 0.19)	0.2	0.11 (0.01 to 0.20)	0.032
**Lifestyle parameters**					
Alcohol intake, units/week, *n* (%)					
None	109 (37)	Reference	n/a	Reference	n/a
< 7 units/week	118 (41)	− 0.09 (− 0.22 to 0.04)	0.2	− 0.07 (− 0.19 to 0.04)	0.2
≥ 7 units/week	64 (22)	− 0.03 (− 0.16 to 0.11)	0.7	− 0.09 (− 0.21 to 0.02)	0.1
Smoking history, *n* (%)	150 (49)	− 0.03 (− 0.09 to 0.14)	0.6	− 0.07 (− 0.17 to 0.03)	0.2
**Transplant-specific characteristics**					
Dialysis before transplantation, *n* (%)	188 (61)	0.16 (0.05 to 0.27)	0.006	0.11 (0.01 to 0.21)	0.025
Living donor, *n* (%)	171 (55)	− 0.17 (− 0.29 to − 0.06)	0.002	− 0.08 (− 0.17 to 0.02)	0.1
Delayed graft functioning, *n* (%)	32 (11)	0.09 (-0.02 to 0.21)	0.1	0.03 (− 0.06 to 0.13)	0.5
Time after transplantation, years^†^	4 [1 to 11]	0.07 (− 0.04 to 0.18)	0.2	0.04 (− 0.05 to 0.14)	0.4
History of rejection(s), *n* (%)	31 (10)	0.07 (− 0.05 to 0.18)	0.2	0.09 (− 0.01 to 0.19)	0.064
Postoperative CMV infection, *n* (%)	44 (15)	0.08 (− 0.03 to 0.20)	0.2	0.04 (− 0.06 to 0.14)	0.4
**Patient reported outcome measurements **					
Feeling of anxiety, *n* (%)	68 (23)	0.01 (− 0.10 to 0.13)	0.8	0.06 (− 0.04 to 0.16)	0.2
Moderate to severe depressive symptoms, *n* (%)	15 (5)	− 0.10 (− 0.21 to 0.02)	0.097	− 0.05 (− 0.15 to 0.05)	0.3
**Laboratory measurements**					
Hemoglobin, g/dL	13.5 ± 1.8	0.03 (− 0.14 to 0.09)	0.7	− 0.15 (− 0.25 to − 0.05)	0.004
Leukocyte count, 10^9^/L	7.5 ± 2.2	0.01 (− 0.10 to 0.13)	0.8	0.02 (− 0.07 to 0.12)	0.6
C-reactive protein, mg/L^‡^	1.9 [0.7 to 4.1]	0.14 (0.03 to 0.25)	0.014	0.11 (0.01 to 0.21)	0.025
Plasma albumin, g/dL	4.3 ± 0.3	− 0.18 (− 0.29 to − 0.07)	0.002	− 0.11 (− 0.21 to − 0.01)	0.025
eGFR, mL/min/1.73m^2^	52 ± 17	− 0.11 (− 0.22 to 0.01)	0.064	− 0.07 (− 0.17 to 0.02)	0.1
**Immunosuppressive drugs**					
Prednisolone, *n* (%)	300 (97)	0.11 (− 0.00 to 0.22)	0.06	0.08 (− 0.02 to 0.17)	0.1
Calcineurin inhibitor, *n* (%)	255 (83)	− 0.08 (− 0.19 to 0.04)	0.2	− 0.01 (− 0.11 to − 0.09)	0.9
Proliferation inhibitor, *n* (%)	267 (86)	− 0.07 (− 0.18 to 0.05)	0.2	− 0.06 (− 0.16 to 0.04)	0.2
mTOR inhibitor, *n* (%)	12 (4)	− 0.05 (− 0.17 to 0.06)	0.4	− 0.04 (− 0.14 to 0.05)	0.4

Normally distributed data are presented as mean ± standard deviation, non-normally distributed data as median [interquartile range], and categorical data as number (valid %). ^†^: Variables were log_2_ transformed to meet assumptions of linear regression analyses. Higher positive standardized beta coefficients indicate slower performance of the 9-hole peg test, and hence worse hand dexterity. Data regarding educational level, alcohol intake, smoking history, history of rejection, postoperative CMV infection, feeling of anxiety and moderate to severe depressive symptoms, were missing in 5 (2%), 18 (6%), 1 (0.3%), 7 (2%), 10 (3%), 7 (2%) and 8 (3%) participants, respectively. Abbreviations: CI, confidence interval; CMV, cytomegalovirus; eGFR, estimated glomerular filtration rate; mTOR, mammalian target of rapamycin; St. β, standardized beta.

## Results

Baseline characteristics and associations with hand dexterity are shown in Table 1.

**Figure 1:**
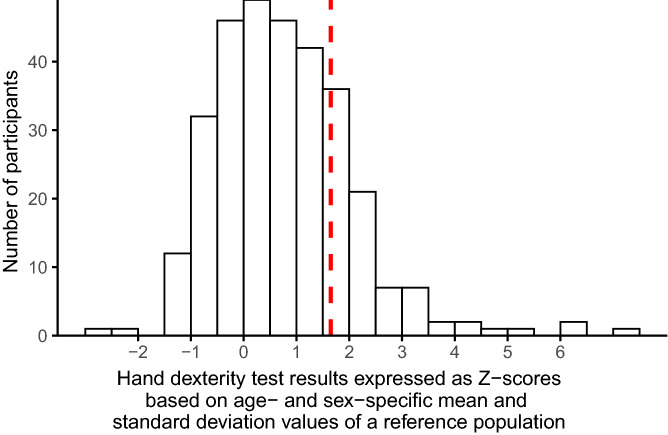
Distribution of Z-scores of the time required to perform the hand dexterity test calculated based on age- and sex-specific mean and standard deviation values of a reference population. The dashed line represents the age- and sex-specific mean + 1.645 times the age- and sex-specific standard deviation of a reference population, which translates to the 95th percentile of the age- and sex-specific reference population^12^. Participants right to this line were defined as having an impaired hand dexterity.

We included 309 KTR (58% male, mean age 56 ± 13 years). Median time after transplantation was 4 [1–11] years, 61% underwent dialysis before transplantation and mean estimated glomerular filtration rate (eGFR) was 52 ± 17 ml/min/1.73m^2^. More extensive transplant specific characteristics and associations with hand dexterity are presented in Supplementary Table S1. Mean time to complete the hand dexterity test was 23.9 ± 4.7 s. Males performed the test slower compared to females (25.0 ± 4.5 vs. 22.3 ± 4.5 s). Among the 309 KTR, 71 (23%) KTR performed the hand dexterity test slower than the age- and sex-specific mean value plus 1.645 times the age- and sex-specific standard deviation of a population-based sample of 3936 mainly Caucasian subjects with a wide age range, and were defined as having an impaired hand dexterity (see Fig. 1). By definition, this implies that the cut-off for hand dexterity is at the 95th percentile of the age and sex-specific reference population.

### Associations with hand dexterity

**Figure 2:**
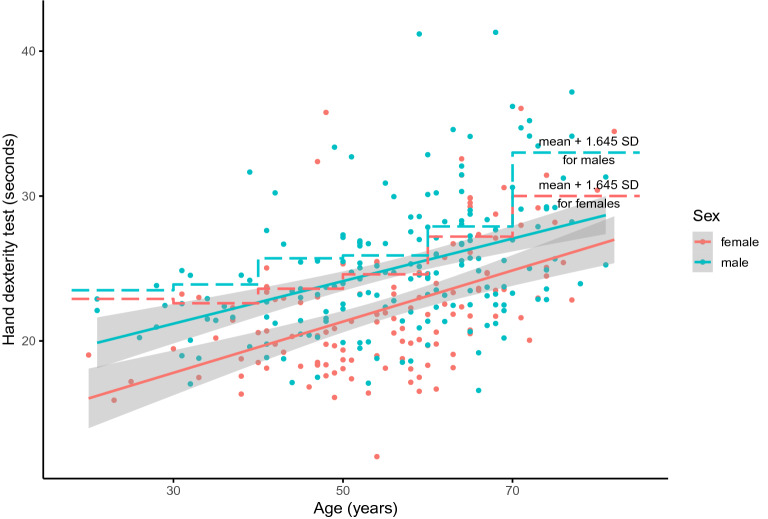
Scatterplot of age and the time to succeed the hand dexterity test, presented per sex. The solid lines represent the association between age and hand dexterity per sex, and the shaded area represents its 95% confidence interval. The dashed lines represent the age- and sex-specific mean + 1.645 times the age- and sex-specific standard deviation of a reference population, stratified by sex^12^. As such, they represent the 95th percentile of an age and sex-specific reference population. Participants above the dashed lines were defined as having an impaired hand dexterity.

In univariable regression analyses, male sex and higher age were associated with longer time required for performance of the 9-hole peg test, which indicates worse hand dexterity (standardized beta (st. β) 0.29, 95%CI 0.18 to 0.40, *p* < 0.001 and st. β 0.44, 95%CI 0.34 to 0.54, *p* < 0.001, respectively). A graphical representation of the association between age and hand dexterity, stratified for sex, is shown in Fig. 2. KTR above the dashed line were regarded as having an impaired hand dexterity.

In further analyses adjusted for sex and age, the most strongly associated variables with hand dexterity were a high educational level (st. β − 0.18, 95%CI − 0.29 to -0.07, *p* < 0.001), which was associated with better hand dexterity, and diabetic kidney disease as primary disease (st. β 0.17, 95%CI 0.06 to 0.28, *p* = 0.003) or suffering from diabetes at time of inclusion (st. β 0.17, 95%CI 0.08 to 0.27, *p* < 0.001), which were both associated with worse hand dexterity.

Among other variables, higher body mass index, anemia and higher C-reactive protein were associated with worse hand dexterity (*p* = 0.025, *p* = 0.032 and *p* = 0.025), whereas dialysis before transplantation, higher hemoglobin and higher plasma albumin were associated with better hand dexterity (*p* = 0.025, *p* = 0.004 and *p* = 0.025). Regarding type of dialysis before transplantation, hemodialysis was associated with worse hand dexterity (*p* = 0.028), while no association with peritoneal dialysis was observed (*p* = 0.1).

Statistically significant effect modification by sex was observed for the associations of diabetes and time since transplantation with hand dexterity (*p* = 0.024 and *p* = 0.023, respectively). In sex-stratified analyses with adjustment for age, diabetes was associated with poor hand dexterity in females (st. β 0.30, 95%CI 0.16 to 0.45, *p* < 0.001), but not in males (st. β 0.09, 95%CI − 0.05 to 0.22, *p* = 0.2). In addition, longer time since transplantation was associated with worse hand dexterity in females (st. β 0.16, 95%CI 0.01 to 0.32, *p* = 0.039), but not in males (st. β − 0.04, 95%CI − 0.17 to 0.10, *p* = 0.6). No effect modification by age and kidney function were observed.

### Measures of physical capacity and ADL

The Timed-Up-and-Go (TUG) test was performed in 7.1 ± 2.0 s, the Five Time Sit To Stand (FTSTS) test in 11.7 ± 3.6 s and the 4-m (4 m) walk test in 3.4 ± 1.3 s. Longer time required to perform these tests indicates worse physical capacity. Fourteen (5%) KTR were unable to perform these physical tests because of incapability to walk safely or walking caused too much pain. Twenty-one (7%) KTR reported limitations in self-care (Supplementary Table S2). Univariable regression analyses showed that worse hand dexterity was associated with more time required to perform the TUG test (st. β 0.36, 95%CI 0.24 to 0.48, *P* < 0.001), the FTSTS test (st. β 0.34, 95%CI 0.22 to 0.45, *P* < 0.001), and the 4 m walk test (st. β 0.26, 95%CI 0.14 to 0.38, *P* < 0.001), all consistent with worse physical capacity. In addition, poor hand dexterity was associated with a higher risk of physical inability to perform the physical assessments (odds ratio (OR) per 5 s increment 2.19, 95%CI 1.34 to 3.59, *P* = 0.002) and self-care limitations (OR per 5 s increment 2.47, 95%CI 1.61 to 3.80, *P* < 0.001). All associations remained statistically significant after cumulative adjustment for potential confounders in model 2, as shown in Table 2. No effect modifications were observed.

**Table 2:** Results of regression analyses with hand dexterity as independent variable and measures of daily functioning as dependent variable.

Physical capacity and ADL	Crude			Model 1			Model 2		
Activities of daily living	*N*	St. β (95% CI)	*P*	*N*	St. β (95% CI)	*P*	*N*	St. β (95% CI)	*P*
Timed-up-and-go test, s^‡^	250	0.36 (0.24 to 0.48)	< 0.001	250	0.35 (0.22 to 0.49)	< 0.001	246	0.29 (0.16 to 0.43)	< 0.001
Five time sit to stand test, s^‡^	258	0.34 (0.22 to 0.45)	< 0.001	258	0.35 (0.21 to 0.48)	< 0.001	255	0.24 (0.11 to 0.38)	< 0.001
4-m walk test, s^‡^	263	0.26 (0.14 to 0.38)	< 0.001	263	0.25 (0.11 to 0.38)	< 0.001	259	0.18 (0.04 to 0.32)	0.012

	*N*	OR (95% CI) ^†^	*P*	*N*	OR (95% CI) ^†^	*P*	*N*	OR (95% CI) ^†^	*P*
Physical inability to perform physical assessments	309	2.19 (1.34 to 3.59)	0.002	309	2.92 (1.62 to 5.28)	< 0.001	303	2.51 (1.12 to 5.59)	0.025
Self-care limitations	309	2.47 (1.61 to 3.80)	< 0.001	309	2.90 (1.75 to 4.80)	< 0.001	303	4.11 (2.02 to 8.37)	< 0.001

Societal participation	*N*	St. β (95% CI)	*P*	*N*	St. β (95% CI)	*P*	*N*	St. β (95% CI)	*P*
Frequency score	265	− 0.35 (− 0.46 to − 0.23)	< 0.001	265	− 0.30 (− 0.43 to − 0.17)	< 0.001	260	− 0.19 (− 0.32 to − 0.05)	0.008
Restriction score	269	− 0.25 (− 0.37 to − 0.13)	< 0.001	269	− 0.35 (− 0.48 to − 0.22)	< 0.001	262	− 0.27 (− 0.41 to − 0.14)	< 0.001
Satisfaction score	267	− 0.03 (− 0.15 to 0.09)	0.6	267	− 0.13 (− 0.27 to 0.01)	0.07	263	− 0.05 (− 0.20 to 0.09)	0.5

Model 1: adjusted for age and sex. Model 2: model 1 additionally adjusted for educational level, body mass index, diabetes, dialysis before transplantation, time since transplantation, hemoglobin, C-reactive protein and plasma albumin. ^†^: Per 5 s increment of the hand dexterity test. ^‡^: Reasons for not performing the physical assessments were an inability to walk safely or walking caused too much pain (*n* = 14), or because of time constraints (*n* = 28). Results are presented in standardized beta (st. β) coefficients for linear regression analyses and in odds ratios (OR) for logistic regression analyses, with 95% confidence intervals (95%CI).

### Measures of societal participation

Unitless scores for frequency, restriction and satisfaction components of societal participation were 31.9 ± 11.4, 87.2 ± 18.1 and 79.0 ± 16.1, respectively (Supplementary Table S2). Higher scores reflect better societal participation. In univariable regression analyses, poorer hand dexterity was associated with a lower frequency of societal participation (st. β − 0.35, 95%CI − 0.46 to − 0.23, *P* < 0.001) and more restrictions in societal participation (st. β − 0.25, 95%CI − 0.37 to − 0.13, *P* < 0.001). Both associations remained statistically significant after cumulative adjustment for potential confounders in model 2. No association was present between hand dexterity and satisfaction of societal participation, as shown in Table 2. No effect modifications were found.

### Health-related quality of life

Unitless scores for Physical Component Scale (PCS) and Mental Component Scale (MCS) of HRQoL were 70.6 ± 21.9 and 77.3 ± 17.1, respectively (Supplementary Table S2). Higher scores reflect better HRQoL. Poorer hand dexterity was associated with worse physical HRQoL (PCS: st. β − 0.24, 95%CI − 0.35 to − 0.13, *P* < 0.001), independent of potential confounders. No association was present between hand dexterity and mental HRQoL, as shown in Table 3. In addition, we observed no effect modifications.

**Table 3:** Analyses with hand dexterity as potential determinant of health-related quality of life.

SF-36	*N*	Health-related quality of life			
		Physical component scale		Mental component scale	
		St. β (95% CI)	P	St. β (95% CI)	*P*
Crude	309	− 0.24 (− 0.35 to − 0.13)	< 0.001	− 0.05 (− 0.17 to 0.06)	0.3
Model 1	309	− 0.29 (− 0.41 to − 0.16)	< 0.001	− 0.15 (− 0.28 to − 0.03)	0.019
Model 2	303	− 0.18 (− 0.30 to − 0.05)	0.007	− 0.08 (− 0.22 to 0.06)	0.3

Model 1: adjusted for sex and age. Model 2: model 1 additionally adjusted for educational level, body mass index, diabetes, dialysis before transplantation, time since transplantation, hemoglobin, C-reactive protein and plasma albumin. Results are presented in standardized beta (st. β) coefficients with 95% confidence intervals (95%CI). Abbreviations: SF-36, Short Form-36; st. β, standardized beta; CI, confidence interval.

### Sensitivity analyses

Characteristics of KTR without impaired hand dexterity and KTR with impaired hand dexterity are presented in Supplementary Table S3. In logistic regression analyses with impaired hand dexterity as dichotomous outcome, most of the associations of baseline variables that were present with hand dexterity as a continuous variable remained.

## Discussion

To best of our knowledge, this study is the first to describe hand dexterity in KTR. Our data show that an impaired interplay between sensory and motor function, as assessed by hand dexterity, is prevalent, and that impaired hand dexterity might be a powerful indicator of diminished overall patient health. Worse hand dexterity was associated with worse performance for almost all measures of physical capacity, ADL and societal participation, independent of potential confounders. In addition, hand dexterity was independently associated with physical HRQoL. These results provide new insights into a hitherto overlooked potential determinant of daily functioning in KTR, and may therefore be an important target for improvement of HRQoL.

We hypothesized that hand dexterity among KTR is impaired, due to acquired damage to the nervous system that may cause an impaired interplay between sensory and motor function. Indeed, our findings confirm this hypothesis, since 23% of the KTR performed the hand dexterity test in more time than > 95th percentile of the normal distribution, based on age- and sex-specific reference values^12^. Thus, the rate of impairment among KTR is more than four times higher compared to a reference population. Besides patients with neurological diseases or hand diseases, a decreased hand dexterity has also been reported in patients suffering from mild or moderate chronic pulmonary disease, and in the elderly, in whom worse hand dexterity was associated with low cognitive performance^11,13,14^.

Our study confirms that hand dexterity is age and sex-dependent^12^. In addition, the presence of diabetes was associated with poor hand dexterity in our population, since diabetic nephropathy as primary renal disease and diabetes at time of inclusion in females were both associated with worse hand dexterity. These associations can likely be attributed to the well-established neuropathic effects of diabetes^15^. Similar associations between diabetes and impaired hand dexterity have been reported in patients with diabetic neuropathy and, in especially the older, insulin-treated, patients with type 2 diabetes^16–18^.

Hemodialysis before transplantation was another determinant of hand dexterity. It is well-known that patients on dialysis frequently have neurological complications, as a result of an uremic state in relation to their kidney failure, or as a result of dialysis therapy^5,6^. Our findings suggest that neurological complications in the pre-transplant period continue even years after kidney transplantation, highlighting the importance of effective management strategies in the pre-transplant period to prevent neurological complications. Additionally, our finding that time after transplantation was also associated with hand dexterity in females, independent of age, suggests that the neurological damage progresses after transplantation. Although immunosuppressive medication is known for its neurotoxic effects, we found no association between immunosuppressive medication use and hand dexterity, which could potentially be related to low variation in use of immunosuppressive medication in our cohort: almost all patients used corticosteroids and the large majority used calcineurin inhibitors^7^. Another possibility is that this association reflects greater time of living with a kidney disease, since patients with a longer disease duration may also have had more time since kidney transplantation. The associations of higher body mass index, anemia, higher C-reactive protein and lower plasma albumin with longer time required for performance of the 9-hole peg test suggest that overall health seems to be a major determinant of hand dexterity.

Our study shows that hand dexterity is strongly positively associated with daily functioning in KTR. This is in agreement with studies performed in elderly, which showed that poor hand dexterity is associated with cognitive impairment and poor executive function, which mainly relates to of attention, planning, judgment, working memory, inhibition and task flexibility^11^. These are all essential for daily functioning. This raises the hypothesis that poor hand dexterity among KTR, and its associations with impaired daily functioning, could be the result of a diminished functioning of the (pre)frontal areas in the brain. These areas are closely involved in executive functions^19,20^. The association between hand dexterity and physical HRQoL signified clinical relevance, and signifies the importance of a well-functioning interplay between sensory and motor function of KTR.

We may speculate that improvement of hand dexterity may improve HRQoL, especially given the indispensable role of the hands and hand function for self-care and societal participation. Hand dexterity can be improved using simple home-based training, for example by means of finger tapping, crossing circles on a sheet, turning discs and modeling clay^21–23^. However, we cannot exclude that the associations of hand dexterity with outcomes are confounded by cognitive function. Future interventional studies are needed to assess the effect of improving hand dexterity among KTR on outcome measures, such as societal participation.

Our results suggest that hand dexterity may be a powerful indicator of diminished health among KTR. Since evaluation of (interventions to improve) daily functioning can be hard in clinical practice, partly due to the limited time in the outpatient clinic, hand dexterity assessments, which are easy, quick and objective, might be useful in the evaluation of daily functioning. Therefore, more research is needed to assess its potential in clinical practice and research.

Strengths of this study are the extensive availability of clinical and biochemical data, together with questionnaire data of stable KTR in the outpatient clinic, allowing us to adjust for many potential confounders. A main limitation is that, due to the observational study design, no conclusions regarding causal relationships can be drawn. Another limitation is that most of the recipients followed the same immunosuppressive regimen, which makes our population less suitable to study effects of immunosuppressive medication on hand dexterity. Unfortunately, there is currently no consensus on the definition of impaired hand dexterity^24^. We applied a definition of impaired hand dexterity using a cut-off value at the age- and sex-specific 95th percentile of the general population. This method allows for easy interpretation of the study results, and direct comparison of prevalences between the general population and the KTR population. The applied definition allows for providing a good impression of the size of the problem of impaired hand dexterity among KTR. Other studies are necessary to determine whether other definitions of impaired hand dexterity are more sensitive and specific to identify an impaired interplay between sensory and motor function. Finally, although hand dexterity was assessed in a subset of KTR which was established through randomization to an assessment path in which physical function tests rather than cognitive tests were performed, we cannot exclude the possibility of selection bias. One reason is that the patients are a subset of a larger group of 1215 KTR of which 19% declined participation in the study. Moreover, in subsets of patients the physical function tests could not be performed because of time constraints.

## Conclusions

In conclusion, impaired interplay between sensory and motor function, as assessed by hand dexterity, is prevalent among KTR. In addition, poor hand dexterity was associated with impaired daily functioning, and limited physical HRQoL. The impaired interplay between sensory and motor function may therefore be an important, hitherto overlooked, phenomenon in KTR. Our results highlight the importance of effective management strategies in the pre- and posttransplant period to prevent neurological complications.

## Methods

### Study population

We used data from the ongoing, prospective, TransplantLines Biobank and Cohort Study (ClinicalTrials.gov identifier: *NCT03272841*), which has started in June 2015 at the University Medical Center Groningen (UMCG) in the Netherlands. All solid organ transplant patients and donors (aged ≥ 18 years) were invited to participate. All participants gave written informed consent on enrolment. The participation rate of this cohort was 81%. The study was approved by the Institutional Review Board of the UMCG (METc 2014/077), adheres to the UMCG Biobank Regulation, and is in accordance with the WMA Declaration of Helsinki and Declaration of Istanbul; no organs were procured from prisoners^25^. All participants were transplanted in the UMCG and/or were treated in the outpatient clinic of the UMCG at time of study inclusion. For current analyses, we included stable KTR with a functioning allograft approximatly 1 year or longer after transplantation with available data regarding hand dexterity (*n* = 311). Two participants were exluded due to severe complaints of polyneuropathy and carpal tunnel syndrome. The inclusion period was between June 2015 and January 2021. A consort flow diagram is presented in Supplementary Fig. S1.

### Hand dexterity assessment

Hand dexterity was assessed using the 9-hole peg test (Sammons, Preston Rolyan, Chicago, Illinois), which is the gold standard metric to assess manual hand dexterity^24^. Participants were instructed to insert, one peg at time, nine pegs into the nine holes, and consequently remove all pegs, one peg at time, with one hand, as quickly as possible. Time from picking up the first peg to laying down the last peg was measured in seconds, using a digital clock. The test was first performed with the dominant hand (88% was right-handed and 12% was left-handed), and repeated with the non-dominant hand. The mean time in seconds of both measurements was used. A higher value indicates longer time until completion, and thus worse hand dexterity. Reference values of the 9-hole peg test of 3936 mainly Caucasian healthy individuals with a wide age range were presented by Wang et al., who provided a mean and standard deviation per sex, hand, and age category. We defined impaired hand dexterity as a duration of the 9-hole peg test more than 1.645 age- and sex-specific standard deviations above the age- and sex-specific mean of the reference population, where the means and standard deviations were averaged for both hands (Supplementary Table S4)^12^. By definition, this implies that the cut-off for hand dexterity is at the 95th percentile of the age- and sex-specific reference population.

### Assessment of daily functioning

Physical capacity and the ability to perform ADL were assessed with the TUG test and FTSTS test, which both assess functional mobility and require coordination, balance and strength, together with the 4 m walk test, which assesses locomotion and gait speed^25–28^. In addition, a trained researcher scored whether the participant was unable to perform the physical tests because of incapability to walk safely or because walking caused too much pain, which was based on the clinical judgment of the researcher and with verification by consultation of the participant. To further assess ADL, participants were asked if they experience limitations in self-care. Societal participation was evaluated using the 32-item Utrecht Scale for Evaluation of Rehabilitation-Participation (USER-P) questionnaire, resulting in a frequency, restrictions, and satisfaction score of societal participation^29^. For the frequency score, participants were asked how many hours per week they do (un)paid work, study or do household chores, and how many times they did activities in the past four weeks, such as sports, daytrips and visiting friends, among others. For the restriction score, participants were asked whether they were restricted by their medical status in daily activities, such as work, their relationship with a partner or having contact with other people by phone or computer, among others. For the satisfaction score, participants were asked how satisfied they are with their work/education, how they move outdoor and their contacts with friends or acquaintances, among others. For the questions regarding restrictions and satisfaction of societal participation, there was an answer option ‘does not apply’. Higher scores reflect better societal participation.

### Assessment of HRQoL

HRQoL was assessed using the Dutch translated Short Form 36 (SF-36) health survey. The PCS was calculated by averaging the scores of physical health, role limitation due to impairment of physical health, general health and pain items. The MCS was calculated by using the mean score of emotional well-being, role limitations due to emotional problems, impaired vitality and impaired social functioning^30,31^. Higher PCS and MCS scores indicate better HRQoL.

### Assessement of covariables

All measurements were performed during a visit to the outpatient clinic. Blood was drawn shortly before this visit after a fasting period of 8 to 12 h; laboratory parameters were measured using routine laboratory methods. Demographic data, transplant specific data and medication use were extracted from the patient’s record. Medication use was consequently verified with the participant. Data regarding alcohol intake and smoking history were gathered by questionnaires. eGFR was calculated with the Chronic Kidney Disease Epidemiology Collaboration equation, diabetes was defined according to criteria of the American Diabetes Association and anemia according to criteria of the WHO^32–34^. Anxiety was assessed using the State-Trait Anxiety Inventory-6 Item short form (STAI-6). A cut-off score ≥ 40 was used to identify a feeling of anxiety^35,36^. Symptoms of depression were assessed using the Patient Health Questionnaire-9 (PHQ-9). Moderate to severe depressive symptoms were defined as a score of ≥ 10^37^.

### Statistical Analyses

Data distributions were visually assessed using histograms and Q-Q plots. Normally distributed data were presented as mean ± standard deviation, non-normally distributed data as median [interquartile range], and categorical data as number (valid %). Differences between groups were assessed using independent T-tests, Mann–Whitney U test and Chi-square tests. Associations between baseline variables with hand dexterity were analyzed using linear regression analyses. After crude analyses, we repeated these analyses with adjustments for sex and age. Presence of effect modification by age, sex, and kidney function were assessed by adding interaction terms into the models.

Potential associations of hand dexterity with daily functioning and HRQoL were investigated using linear and logistic regression analyses with TUG-test, FTSTS test, 4 m walk test, physical inability to perform physical assessments, limitations in self-care, USER-P outcomes (frequency, restriction, and satisfaction score), PCS and MCS as dependent variable and hand dexterity as independent variable. After crude analyses, we adjusted for the potential confounders age and sex in model 1, and in model 2 additionally for educational level, body mass index, diabetes, dialysis before transplantation, time since transplantation, hemoglobin, C-reactive protein and plasma albumin. Again, we assessed if effect modifications by age, sex or kidney function were present.

Strengths and directions of associations derived from linear regression analyses are presented as st. β coefficients, which represent the number of standard deviations a dependent variable changes per standard deviation increase of the independent variable. In the results of analyses to assess associations between baseline characteristics and hand dexterity, higher positive st. β coefficients indicate slower performance and hence worse hand dexterity. Logistic regression analyses were presented as OR. In regression analyses, a two-sided *P* < 0.05 was regarded as statistically significant. All statistical analyses were performed using the Statistical Package for the Social Sciences (SPSS) version 25.0, and data were presented using R version 3.5.2. The consort flow diagram was created using Visio Professional 2021.

## Supplementary Information

Supplementary Information.

## Data Availability

All data presented in this study can be made available by the data manager of the Transplantlines study, by mailing to datarequest.transplantlines@umcg.nl.
