# Supplementary material for: Hand dexterity, daily functioning and health-related quality of life in kidney transplant recipients
Source: Sci Rep. 2022 Sep 28;12:16208. doi: 10.1038/s41598-022-19952-5 (PMC9519570; doi:10.1038/s41598-022-19952-5)
Supplement: Supplementary file 1 — Supplementary Information. [file 41598_2022_19952_MOESM1_ESM.docx]

**Supplementary information of the manuscript entitled: Hand Dexterity, Daily Functioning and Health-Related Quality of Life in Kidney Transplant Recipients**

Tim J Knobbe, MD, Daan Kremer, MD, Michele F Eisenga, MD, PhD, Eva Corpeleijn, PhD, Coby Annema, PhD, Joke M. Spikman, PhD, Transplantlines Investigators, Gerjan Navis, MD, PhD, Stefan P Berger, MD, PhD, Stephan JL Bakker, MD, PhD


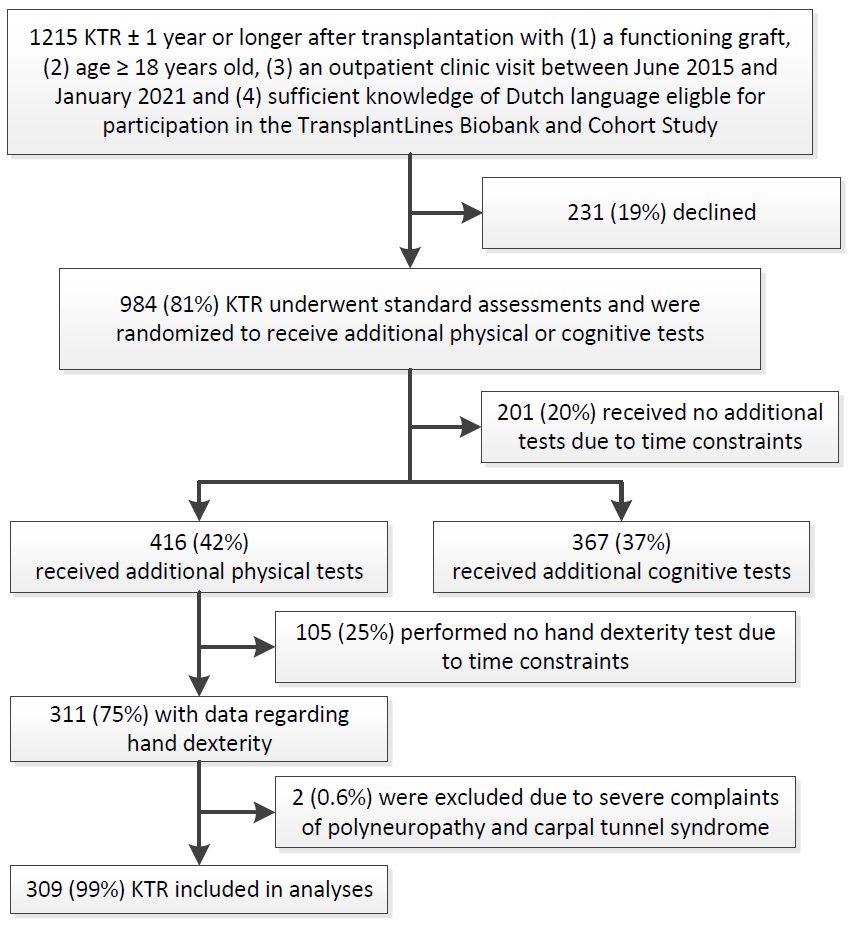


**Supplementary Figure S1.** Consort flow diagram. Abbreviations: KTR, kidney transplant recipients.

**Supplementary Table S1.** More extensive transplant specific characteristics in addition to characteristics presented in Table 1.

| **Transplant-specific characteristics** | | **Total population**  **N=309** | **Results of linear regression analyses** | | | |
| --- | --- | --- | --- | --- | --- | --- |
|  |  |  | **Univariable analyses** | | **Analyses adjusted for sex and age** | |
|  |  |  | **St. β (95% CI)** | **P** | **St. β (95% CI)** | **P** |
| **Recipient Characteristics** | |  |  |  |  |  |
| Dialysis type before Tx, *n* (%) | |  |  |  |  |  |
|  | No dialysis before Tx | 121 (27) | *reference* |  | *reference* |  |
|  | Hemodialysis | 104 (34) | 0.15 (0.03 to 0.28) | 0.015 | 0.12 (0.01 to 0.23) | 0.028 |
|  | Peritoneal dialysis | 84 (27) | 0.14 (0.02 to 0.26) | 0.027 | 0.08 (-0.02 to 0.19) | 0.1 |
| Positive CMV status before Tx, *n* (%) | | 153 (52) | 0.13 (0.01 to 0.24) | 0.029 | 0.09 (-0.01 to 0.19) | 0.078 |
| **Donor characteristics** | |  |  |  |  |  |
| Donor age, years | | 50 ± 14 | 0.02 (-0.10 to 0.13) | 0.8 | -0.08 (-0.18 to 0.02) | 0.096 |
| Donor type, *n* (%) | |  |  |  |  |  |
|  | Living donor | 171 (56) | *reference* |  | *reference* |  |
|  | Donation after circulatory death | 54 (18) | 0.12 (0.00 to 0.23) | 0.046 | 0.03 (-0.08 to 0.13) | 0.6 |
|  | Donation after brain death | 82 (27) | 0.17 (0.06 to 0.29) | 0.003 | 0.09 (-0.01 to 0.19) | 0.073 |
| Positive CMV status of the donor, *n* (%) | | 141 (47) | 0.04 (-0.08 to 0.15) | 0.5 | 0.01 (-0.09 to 0.11) | 0.9 |
| **Transplant characteristics** | |  |  |  |  |  |
| 1^st^ warm ischemia time, minutes | |  |  |  |  |  |
|  | Donation after circulatory death | 16.8 ± 12.9 | -0.16 (-0.44 to 0.13) | 0.3 | -0.11 (-0.38 to 0.16) | 0.4 |
|  | Living donor | 3.5 ± 3.2 | -0.06 (-0.22 to 0.09) | 0.4 | -0.09 (-0.21 to 0.04) | 0.2 |
| Cold ischemia time, hours | |  |  |  |  |  |
|  | Deceased donor | 15.7 ± 14.5 | 0.02 (-0.15 to 0.19) | 0.8 | 0.04 (-0.12 to 0.20) | 0.6 |
|  | Living donor | 2.7 ± 0.5 | 0.11 (-0.04 to 0.27) | 0.2 | 0.06 (-0.06 to 0.19) | 0.3 |
| 2^nd^ warm ischemia time, minutes | | 40.8 ± 11 | -0.04 (-0.15 to 0.08) | 0.5 | -0.03 (-0.13 to 0.07) | 0.5 |
| Postoperative CMV infection, *n* (%) | |  |  |  |  |  |
|  | No CMV infection | 255 (85) | *reference* |  | *reference* |  |
|  | Primary infection | 23 (8) | 0.06 (-0.06 to 0.18) | 0.3 | 0.03 (-0.07 to 0.13) | 0.6 |
|  | Secondary infection | 21 (7) | 0.06 (-0.05 to 0.18) | 0.3 | 0.03 (-0.07 to 0.13) | 0.6 |

Impaired hand dexterity was defined as a duration of the 9-hole peg test more than 1.645 age- and sex specific standard deviations above the age- and sex-specific mean (>95^th^ percentile) from a reference population, consisting of 3936 subjects with a wide age range, as presented by Wang *et al*^12^. Data regarding history of rejection, CMV status of the recipient before transplantation, donor type, CMV status donor, 1^st^ warm ischemia time, cold ischemia time, 2^nd^ warm ischemia time, and postoperative CMV infection were missing in 7 (2%), 14 (5%), 2 (1%), 8 (3%), 9 (3%), 21 (7%) and 9 (3%), and 10 (3%) participants, respectively. Abbreviations: CI, confidence interval; CMV, cytomegalovirus; St. β, standardized beta; Tx, transplantation.

**Supplementary Table S2.** Measures of daily functioning and health-related quality of life.

|  | | **Total population**  **N=309** |
| --- | --- | --- |
| **Physical activity and activities of daily living** | |  |
|  | Timed up and go test, s | 7.1 ± 2.0 |
|  | Five time sit to stand test, s | 11.7 ± 3.6 |
|  | 4 meter walk test, s | 3.4 ± 1.3 |
|  | Physical inability to perform physical assessments, *n* (%) | 14 (5) |
|  | Limitations in self-care, *n* (%) | 21 (7) |
| **Societal participation** | |  |
|  | Frequency score | 31.9 ± 11.4 |
|  | Restrictions score | 87.2 ± 18.1 |
|  | Satisfaction score | 79.0 ± 16.1 |
| **Health-related quality of life** | |  |
|  | Physical component scale | 70.6 ± 21.9 |
|  | Mental component scale | 77.3 ± 17.1 |

Normally distributed data are presented as means ± standard deviation and categorical data as number (valid %). Data regarding Timed-up-and-go test, sit-to-stand test, 4m walk test, frequency score, the restriction score, satisfaction score was missing in 59 (19%), 51 (17%), 46 (15%), 44 (14%), 40 (13%) and 42 (14%). No data were missing for the variables limitations in self-care and health-related quality of life

**Supplementary Table S3.** Associations with impaired hand dexterity.

|  | | **No impaired hand dexterity**  **N=238 (77%)** | **Impaired hand dexterity**  **N=71 (23%)** | **Results of univariable logistic regression analyses** | |
| --- | --- | --- | --- | --- | --- |
| **Demographics** | |  |  | **OR**^†^ **(95% CI)** | **P** |
| Male sex, *n* (%) | | 132 (56) | 48 (68) | 1.68 (0.96 to 2.93) | 0.070 |
| Age, years | | 55 ± 13 | 57 ± 13 | 1.19 (0.90 to 1.56) | 0.2 |
| Educational level, *n* (%) | |  |  |  |  |
|  | Low | 80 (34) | 34 (49) | 1 (reference) |  |
|  | Medium | 91 (39) | 23 (33) | 0.59 (0.32 to 1.09) | 0.094 |
|  | High | 64 (27) | 12 (17) | 0.44 (0.21 to 0.92) | 0.029 |
| Caucasian, *n* (%) | | 231 (97) | 66 (83) | 0.40 (0.12 to 1.30) | 0.1 |
| Body mass index, kg/m^2^ | | 27 ± 5 | 29 ± 5 | 1.29 (1.00 to 1.66) | 0.050 |
| Primary kidney disease, *n* (%) | |  |  |  |  |
|  | Unknown | 35 (15) | 11 (16) | 1 (reference) |  |
|  | Inflammatory disease | 78 (33) | 25 (35) | 1.02 (0.45 to 2.30) | 1.0 |
|  | Congenital and hereditary kidney disease | 74 (31) | 9 (13) | 0.49 (0.15 to 1.02) | 0.055 |
|  | Kidney vascular disease, excl. vasculitis | 20 (8) | 7 (10) | 1.11 (0.37 to 3.33) | 0.8 |
|  | Diabetic kidney disease | 8 (3) | 12 (17) | 4.77 (1.55 to 14.66) | 0.006 |
|  | Other | 23 (10) | 7 (10) | 0.97 (0.33 to 2.86) | 1.0 |
| Diabetes, *n* (%) | | 56 (24) | 30 (42) | 2.38 (1.36 to 4.16) | 0.002 |
| Anemia, *n* (%) | | 69 (29) | 27 (38) | 1.50 (0.86 to 2.62) | 0.2 |
| **Lifestyle parameters** | |  |  |  |  |
| Alcohol intake, units/week, *n* (%) | |  |  |  |  |
|  | None | 78 (35) | 31 (44) | 1 (reference) |  |
|  | <7 units/week | 92 (42) | 26 (37) | 0.71 (0.39 to 1.30) | 0.3 |
|  | ≥7 units/week | 50 (23) | 14 (20) | 0.70 (0.34 to 1.45) | 0.3 |
| Smoking history, *n* (%) | | 119 (50) | 31 (44) | 0.77 (0.45 to 1.31) | 0.3 |
| **Transplant-specific characteristics** | |  |  |  |  |
| Dialysis before Tx, *n* (%) | | 136 (57) | 52 (73) | 2.05 (1.14 to 3.68) | 0.016 |
| Dialysis type before Tx, *n* (%) | |  |  |  |  |
|  | No dialysis before Tx | 102 (43) | 19 (27) | 1 (reference) |  |
|  | Hemodialysis | 72 (30) | 32 (45) | 2.39 (1.25 to 4.54) | 0.008 |
|  | Peritoneal dialysis | 64 (27) | 20 (28) | 1.68 (0.83 to 3.38) | 0.1 |
| Donor age, years | | 51 ± 13 | 49 ± 15 | 0.86 (0.66 to 1.12) | 0.2 |
| Donor type, *n* (%) | |  |  |  |  |
|  | Living donor | 135 (57) | 36 (51) | 1 (reference) |  |
|  | Donation after circulatory death | 38 (16) | 16 (23) | 1.58 (0.79 to 3.15) | 0.2 |
|  | Donation after brain death | 63 (27) | 19 (27) | 1.13 (0.60 to 2.13) | 0.7 |
| 1^st^ warm ischemia time, minutes | |  |  |  |  |
|  | Donation after circulatory death | 16.2 ± 14.2 | 18.2 ± 9.5 | 1.16 (0.66 to 2.05) | 0.6 |
|  | Living donor | 3.7 ± 3.5 | 2.9 ± 1.5 | 0.58 (0.28 to 1.18) | 0.1 |
| Cold ischemia time, hours | |  |  |  |  |
|  | Deceased donor | 15.6 ± 5.5 | 15.8 ± 7.0 | 1.03 (0.68 to 1.54) | 0.9 |
|  | Living donor | 2.7 ± 0.5 | 2.8 ± 0.6 | 1.23 (0.86 to 1.77) | 0.3 |
| 2^nd^ warm ischemia time, minutes | | 51 ± 13 | 49 ± 15 | 0.94 (0.72 to 1.24) | 0.7 |
| History of rejection(s), *n* (%) | | 21 (9) | 10 (14) | 1.67 (0.75 to 3.75) | 0.2 |
| Delayed graft functioning, *n* (%) | | 23 (10) | 9 (13) | 1.34 (0.59 to 3.05) | 0.5 |
| Time after transplantation, years | | 4 [1 to 11] | 3 [1 to 13] | 1.07 (0.83 to 1.39) | 0.6 |
| Positive CMV status before Tx, *n* (%) | | 110 (48) | 43 (64) | 1.92 (1.09 to 3.37) | 0.023 |
| Positive CMV status of the donor, *n* (%) | | 107 (46) | 141 (47) | 1.09 (0.64 to 1.87) | 0.7 |
| Postoperative CMV infection, *n* (%) | |  |  |  |  |
|  | No CMV infection | 198 (86) | 57 (81) | 1 (reference) |  |
|  | Primary infection | 15 (7) | 8 (11) | 1.85 (0.75 to 4.59) | 0.2 |
|  | Secondary infection | 16 (7) | 5 (7) | 1.09 (0.38 to 3.09) | 0.9 |
| **Patient reported outcome measurements** | | |  |  |  |
| Feeling of anxiety, *n* (%) | | 48 (21) | 20 (30) | 1.61 (0.87 to 2.96) | 0.1 |
| Severe to moderate severe depressive symptoms, *n* (%) | | 11 (5) | 4 (6) | 1.24 (0.38 to 4.03) | 0.7 |
| **Laboratory measurements** | |  |  |  |  |
| Hemoglobin, g/dL | | 13.9 ± 1.8 | 13.9 ± 2.0 | 0.91 (0.70 to 1.18) | 0.5 |
| Leukocyte count, 10^9^/L | | 7.5 ± 2.2 | 7.7 ± 2.3 | 1.07 (0.83 to 1.39) | 0.6 |
| C-reactive protein, mg/L | | 1.9 [0.7 to 3.9] | 2.7 [1.2 to 5.0] | 1.21 (0.95 to 1.54) | 0.1 |
| ­Plasma albumin, g/dL | | 4.4 ± 0.3 | 4.3 ± 3.5 | 0.75 (0.57 to 0.97) | 0.030 |
| eGFR, mL/min/1.73m^2^ | | 52 ± 16 | 48 ± 19 | 0.78 (0.60 to 1.03) | 0.081 |
| **Immunosuppressive drugs** | |  |  |  |  |
| Prednisolone, *n* (%) | | 230 (97) | 70 (99) | 2.43 (0.30 to 19.80) | 0.4 |
| Calcineurin inhibitor, *n* (%) | | 194 (82) | 61 (86) | 1.38 (0.66 to 2.91) | 0.4 |
| Proliferation inhibitor, *n* (%) | | 207 (87) | 60 (85) | 0.82 (0.39 to 1.72) | 0.6 |
| mTOR inhibitor, *n* (%) | | 11 (5) | 1 (0.3) | 0.25 (0.04 to 2.32) | 0.2 |

Impaired hand dexterity was defined as a duration of the 9-hole peg test more than 1.645 age- and sex specific standard deviations above the age- and sex-specific mean (>95^th^ percentile) from a reference population, consisting of 3936 subjects with a wide age range, as presented by Wang *et al*^12^. ^†^: odds ratios are presented per standard deviation increment for continuous variables. Educational level was missing in 5 (2%) of the participants, alcohol intake in 18 (6%), smoking history in 1 (0.3%), history of rejection in 7 (2%), CMV status of the recipient before transplantation in 14 (5%), CMV status donor of 8 (3%), postoperative CMV infection in 10 (3%), feeling of anxiety in 7 (2%) and feeling of moderate to severe depressive symptoms in 8 (3%). Abbreviations: CMV, cytomegalovirus; eGFR, estimated glomerular filtration rate; KTR, kidney transplant recipients; mTOR, mammalian target of rapamycin; OR, odds ratio; SD, standard deviation; Tx, transplantation.

**Supplementary Table S4.** Reference values of the 9-hole peg test and definition of an impaired hand dexterity.

|  | **Males** | |  | **Females** | |
| --- | --- | --- | --- | --- | --- |
| Age | Mean ± standard deviation of the reference population | Impaired hand dexterity­­^1^ |  | Mean ± standard deviation of the reference population | Impaired hand dexterity_­­_^1^ |
| 18-29 years | 19.3 ± 2.6 | >23.5 |  | 19.2 ± 2.3 | >22.9 |
| 30-39 years | 19.9 ± 2.5 | >23.9 |  | 19.0 ± 2.2 | >22.6 |
| 40-49 years | 20.7 ± 3.1 | >25.7 |  | 19.4 ± 2.6 | >23.6 |
| 50-59 years | 21.2 ± 2.9 | >25.9 |  | 19.8 ± 3.0 | >24.6 |
| 60-69 years | 23.0 ± 3.0 | >27.9 |  | 21.3 ± 3.6 | >27.2 |
| 70-85 years | 25.7 ± 4.5 | >33.0 |  | 23.7 ± 3.9 | >30.0 |

^1^: calculated using the formula: age- and sex specific mean plus 1.645 times the age- and sex specific standard deviation of a reference population consisting of 3936 healthy, mainly Caucasian, individuals with a wide age range, of which Wang et al. presented data per hand in the presented age categories ^12^. The mean of the dominant and non-dominant hand was taken to calculate a reference value. Data presented are in seconds.
